# Efficient Resource-Aware Convolutional Neural Architecture Search for Edge Computing with Pareto-Bayesian Optimization

**DOI:** 10.3390/s21020444

**Published:** 2021-01-10

**Authors:** Zhao Yang, Shengbing Zhang, Ruxu Li, Chuxi Li, Miao Wang, Danghui Wang, Meng Zhang

**Affiliations:** School of Computer Science and Engineering, Northwestern Polytechnical University, Xi’an 710072, China; yz70528@mail.nwpu.edu.cn (Z.Y.); zhangsb@nwpu.edu.cn (S.Z.); lrx07@mail.nwpu.edu.cn (R.L.); lichuxi@mail.nwpu.edu.cn (C.L.); miw43@mail.nwpu.edu.cn (M.W.); wangdh@nwpu.edu.cn (D.W.)

**Keywords:** edge computing, neural architecture search, latency profiling model, Pareto-Bayesian optimization

## Abstract

With the development of deep learning technologies and edge computing, the combination of them can make artificial intelligence ubiquitous. Due to the constrained computation resources of the edge device, the research in the field of on-device deep learning not only focuses on the model accuracy but also on the model efficiency, for example, inference latency. There are many attempts to optimize the existing deep learning models for the purpose of deploying them on the edge devices that meet specific application requirements while maintaining high accuracy. Such work not only requires professional knowledge but also needs a lot of experiments, which limits the customization of neural networks for varied devices and application scenarios. In order to reduce the human intervention in designing and optimizing the neural network structure, multi-objective neural architecture search methods that can automatically search for neural networks featured with high accuracy and can satisfy certain hardware performance requirements are proposed. However, the current methods commonly set accuracy and inference latency as the performance indicator during the search process, and sample numerous network structures to obtain the required neural network. Lacking regulation to the search direction with the search objectives will generate a large number of useless networks during the search process, which influences the search efficiency to a great extent. Therefore, in this paper, an efficient resource-aware search method is proposed. Firstly, the network inference consumption profiling model for any specific device is established, and it can help us directly obtain the resource consumption of each operation in the network structure and the inference latency of the entire sampled network. Next, on the basis of the Bayesian search, a resource-aware Pareto Bayesian search is proposed. Accuracy and inference latency are set as the constraints to regulate the search direction. With a clearer search direction, the overall search efficiency will be improved. Furthermore, cell-based structure and lightweight operation are applied to optimize the search space for further enhancing the search efficiency. The experimental results demonstrate that with our method, the inference latency of the searched network structure reduced 94.71% without scarifying the accuracy. At the same time, the search efficiency increased by 18.18%.

## 1. Introduction

In recent years, with the development of machine learning theory and the advancement of embedded devices, IoT devices and mobile phones, the combination of them has great application potential. On the one hand, various sensors (cameras, microphones and GPS) in these devices can generate personalized data with different users in various application scenarios (navigation, positioning and behavior detection [1,2,3,4]). At the same time, with the development of dedicated computing architectures and computing engines, the computing capability of these devices has been significantly improved, so that the data collected by sensors can be processed locally on these devices to meet users’ requirements and guarantee data security. When processing these data collected by sensors into intelligent applications, deep learning algorithms are the best choices. In this case, deep learning algorithms have been applied to machine learning fields, such as Computer Vision (CV) [5,6] and Nature Language Processing (NLP) [7]. It can be foreseen that on-device intelligence or edge intelligence will be ubiquitous.

However, directly deploying state-of-the-art (SOTA) deep neural networks into an edge device still faces many challenges. The most important problem is the conflict between the scales of neural networks and the computation capabilities of devices. As we all know, in order to improve the learning ability of neural networks, wider and deeper network structures [8,9] have been designed, which causes the explosive growth concerning the scale of network parameters. It is impractical to deploy these neural networks in edge devices with limited computation resources, especially in edge computing scenarios with high real-time performance requirements, such as robots and autonomous driving.

In order to resolve the conflict between the scale of the neural network and the computation resources of the devices, some lightweight network structures [10,11,12] have been designed. At the same time, due to the redundancy of parameters in the neural network, some neural network compact algorithms [13,14,15,16,17,18] have also been proposed. Unfortunately, these methods are difficult to design for specific devices and requirements, and only provide a solution to the problem from a general perspective. For example, lightweight structure design provides convolutional operations with fewer computation operations, and according to compact algorithms, there are a lot of redundant parameters in the network. However, when optimizing the oversized network structures, these methods not only require a lot of professional knowledge but also need numerous manual trials and errors to find out the trade-off between the scale of parameters and accuracy.

Neural Architecture Search (NAS) [19,20,21] provides us with a more promising method, without manual interventions. Furthermore, it can automatically search the network structure, such as the operations of each layer and the connections between layers, within a given search space to achieve the best accuracy. Furthermore, in order to balance the accuracy and hardware performance of the network, the methods with hardware performance as one of the searching targets have been put forward [22,23]. However, in these methods, only the hardware performances (inference latency, power consumption and energy consumption) are used to evaluate a network structure, instead of illustrating the resource consumption characteristics of a certain network structure and certain computing operation during the search process. Therefore, it is hard to clarify the impact of every choice of computing operation and layer connections in a network on the hardware performance of the whole network. As a result, the search direction can only be obtained by continuously enumerating the network structures during the search process, instead of changing the search direction clearer, which reduces the search efficiency to a great extent. At the same time, when constructing the search space, the network structure settings and layer operation choices will make a huge difference in the network structure and the scale of parameters, which will further affect the search efficiency.

**Figure 1:**
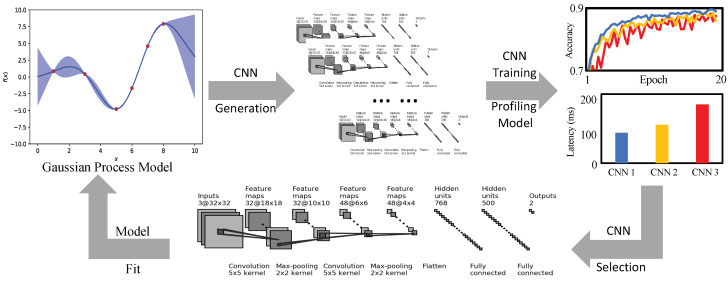
The overview of the proposed method.

Therefore, in order to solve the problems mentioned above, an efficient NAS framework with the ability of hardware resource consumption characteristics awareness is put forward. Based on Bayesian NAS, three strategies are adopted to efficiently search for the neural network featured with high accuracy and can satisfy specific hardware performance requirements. An overview of our work is shown in Figure 1. Firstly, the resource consumptions of any given neural network are described to determine the inference latency of both each operation and the whole network. Secondly, on the basis of the Bayesian NAS method, by introducing the constraints of computation resources, an optimized Bayesian search algorithm is given. Finally, with the help of cell-based structures and lightweight operations, an optimized search space will be applied to the search process.

Our work has the following three main contributions:

- When computing a neural network, the computation resource bottlenecks of an edge device are identified, which will bring more computation overheads and enlarge the inference latency. Then, the computation workloads, memory usage, and inference latency of a neural network are profiled. With the guidance of the profiling model, the hardware performance impactions of each choice during the search process and the inference latency of the sampled networks can be determined.
- An optimized Bayesian search algorithm is proposed by introducing inference latency as one of the search objectives. With the profiling model, we can determine the resource consumptions and computation overheads of each kind of search choice and instruct to adjust the search direction for the purpose of obtaining neural networks with high accuracy and low inference latency, thereby narrowing down the search space and improving the search efficiency.
- Then, the optimized search space with cell-based structures and lightweight operations is further proposed. Therefore, we can reduce the number of networks that may be sampled and the computation workloads to reduce the training overheads during the search process, which can further enhance the search efficiency.

Experimental evaluations are conducted on the MNIST and CIFAR-10 datasets. The results demonstrate that the proposed method can obtain a neural network with high accuracy and low inference latency and help to improve the search efficiency.

## 2. Preliminary

### 2.1. Multi-Objective NAS

NAS is a process that automatically searches for neural networks for any given dataset and task. At present, there are three main methods for searching the structure of a neural network, and they are based on Reinforcement Learning [24,25,26], Genetic Algorithms [27,28,29,30] and Bayesian Optimization [31,32,33], respectively. When the hardware performance is considered in the search process, multi-objective NAS algorithms [34,35,36,37] are proposed. References [34,36] deploy neural networks on real devices, such as mobile phones, to obtain the hardware performance, while [35,37] apply estimation models to obtain the inference latency. When designing multi-objective search algorithms, these methods only use accuracy and hardware performance to evaluate whether the searched neural network meets the requirements. However, they cannot evaluate the impact of every choice during the search process, such as the resource consumptions and computation overheads for the operations and connections of layers. Therefore, it is impossible to obtain a clear direction to guide the search process. Only with numerous network structure sampling, can we get closer to the search target, thereby significantly reducing search efficiency. At the same time, to get the hardware performances, the actual device deployment will bring additional time costs, especially when thousands of neural networks will be sampled during the search process, and thus the overall search efficiency is reduced.

**Figure 2:**
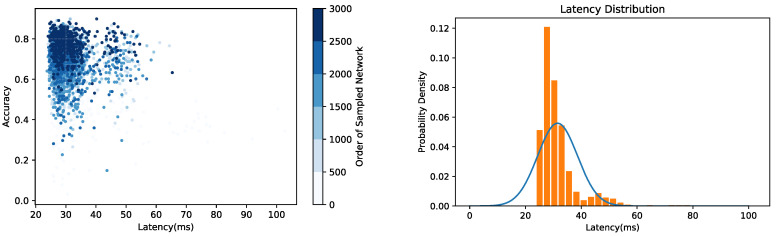
The search results of the preliminary experiment.

Here, a preliminary experiment is conducted on the basis of [24]. To be specific, the accuracy and inference latency of the neural network are set as the search objectives to construct Multi-Objective NAS and confirm the application requirement for inference latency as 30 ms. The neural network search is performed on the CIFAR-10 dataset. Furthermore, the accuracy and inference latency of the 3000 sampled network structures are measured during the search process. The results are shown in Figure 2. Although the accuracy of the network structure is gradually improved and the inference latency converges to the application requirement, the distribution range of the inference latency is still extremely large. It can be seen that when the accuracy of the searched network structure exceeds 80%, the distribution of the inference latency is still mainly between 25 and 35 ms, and the standard deviation (std) of their inference latency is 7.14. In addition to that, the std of the inference latency of the first 2000 sampled network structures is also calculated, and it is 7.80, similar to the inference latency std of the network in which the accuracy exceeds 80%, which means that the inference latency of the searched network with high accuracy is still fluctuating continuously. This result shows that the search process is not constrained by the two-dimensional constraints of accuracy and inference latency. At the same time, it is difficult to reflect a clear trade-off between accuracy and inference latency along the search process. As a result, when the accuracy is gradually enhanced, a large amount of network sampling and training is still required to obtain the optimal network structure, which seriously affects the search efficiency. Therefore, in order to solve this problem, an efficient NAS method is put forward.

Our work is mostly related to [31,32,33], with Bayesian Optimization for NAS that can guide the direction of network morphism (the changes of network structure). With the search direction guiding capability, when we apply our profiling model and assign it with the ability to be aware of the hardware performance, the search space will be further narrowed down with two-dimensional search objectives, and the network morphism will be guided in the direction of high accuracy and low inference latency.

### 2.2. Hardware Performance Evaluation Model

Another relevant topic is the hardware performance evaluation of neural networks. In [38], a runtime analysis model is designed for different deep neural networks (DNN) on variable devices, and it is based on the number of parameters in neural networks. The authors divide the running time of each layer in the DNN into two parts: computing time and communication time. Then, computing time is estimated by floating-point operations and device speed, while communication time is the time of input and output. However, for different devices, their average computing time and communication time are varied. In order to obtain accurate results, a large number of experiments need to be conducted for different DNNs and devices. In [39], the inference latency of the whole network is predicted by sparse polynomial regression while collecting the real running time of different kinds of neural network operations on GPUs. However, these two methods are mainly aimed at GPU platforms, rather than embedded or mobile devices since the inference latency from the perspective of the number of parameters or actual running time does not need to consider the impact of hardware mapping structure or the computation resources on the final results. Therefore, when evaluating the hardware performance of neural networks in embedded devices, mobile devices and edge devices, not only do the hardware mapping structures need to be considered, but also they should consider the computation workloads, memory usages, and data transfer under different computation resources. Only in this way can we obtain a more accurate hardware performance.

## 3. Inference Consumption Profiling for Edge Devices

In order to efficiently search neural networks with high accuracy and low inference latency, the search algorithm with the capability of hardware resource awareness is required, when the ability can help us clarify the changes in resource consumption and the change in inference latency caused by changes in the network structure in each search stage. In this way, the resource consumption characteristics of the operations that can change the network structure will be clarified, which enables us to make adaptive network structure change when facing a low-latency search objective. Therefore, the resource consumptions and computation overheads of the neural network sampled during the search process should be comprehensively evaluated. Specifically, the Convolutional Neural Network (CNN) is taken as our research object based on the given hardware mapping structure, profile the computation workloads, memory usage, and inference latency.

### 3.1. Parallel Mapping Structure

Aiming to accurately profile the inference consumption of a CNN on an edge device, firstly, an appropriate hardware mapping architecture is selected. The accuracy of CNN obtained by NAS exceeds the accuracy of humans in processing the same tasks. However, its structure will be complicated and irregular, which brings extremely high computation complexity. In addition, as different CNNs have different structures, operations, and the corresponding number of parameters, all of these will affect the hardware performance to various degrees. When the computation mode of the neural network in the target hardware is determined, the resource consumptions of complicated network structure and diverse computing operation and their impact on the inference latency can be fully clarified.

In the parallel structure, each layer of the CNN will be mapped onto the chip separately. Furthermore, it is read from the memory when it needs to be calculated and then written back to the memory after the calculation. Compared with the pipeline structure, only one layer is calculated in each round of calculation. In this case, the conflict between computation workload and the memory is moderated. The scale of the neural network will have a limited impact on the hardware performance of the parallel structure. Therefore, the parallel structure is chosen for an edge device.

### 3.2. Theoretical Inference Latency Formulation

Since the parallel structure is adopted for edge devices, when profiling the inference overheads of a CNN, the first thing to be noted is the data movement of any given layer that is the main factor affecting the inference latency, especially on devices with constrained resources. Furthermore, frequent data movement will extend the processing time of the entire neural network architecture. Therefore, before profiling the inference latency, the data movement has to be analyzed when processing a CNN. The data size of the $i_{th}$ layer on CNN can be divided into three parts, namely input data $data_{in}^{\left(i\right)}$, weight data $data_{weight}^{\left(i\right)}$, and output data $data_{out}^{\left(i\right)}$, which can be denoted as follows:

(1) $$data_{in}^{\left(i\right)}=R^{{\left(i\right)}^{2}}\cdot N_{in}^{\left(i\right)}\cdot bitwidth,$$

(2) $$data_{weight}^{\left(i\right)}=K^{{\left(i\right)}^{2}}\cdot N_{in}^{\left(i\right)}\cdot N_{out}^{\left(i\right)}\cdot bitwidth,$$

(3) $$data_{out}^{\left(i\right)}=S^{{\left(i\right)}^{2}}\cdot N_{out}^{\left(i\right)}\cdot bitwidth.$$

The notations are described in Table 1.

Considering the data size and on-chip buffer, if the on-chip buffer is smaller than the data size, the data movement is:

(4) $$IO^{\left(i\right)}=data_{in}^{\left(i\right)}+data_{weight}^{\left(i\right)}\cdot N_{out}^{\left(i\right)}+data_{out}^{\left(i\right)},$$

while, if the on-chip buffer is bigger than the data size, the data movement is:

(5) $$IO^{\left(i\right)}=data_{in}^{\left(i\right)}+data_{weight}^{\left(i\right)}+data_{out}^{\left(i\right)}.$$

After identifying the data movement of each layer, we will elaborate the processing time of each layer and the overall inference latency of a CNN. The processing time of the $i_{th}$ layer can also fall into three parts: computation time $T_{comp}^{\left(i\right)}$, loading time $T_{load}^{\left(i\right)}$ and physical operation time $T_{po}^{\left(i\right)}$. The data of each layer directly affects the computation time, and the number of layers influences the overall latency. There is a positive correlation between the amount of data and computation time. At the same time, in the parallel structure, high parallelism can improve performance by transferring data. Therefore, it is negatively related to the computation time. The load time is determined by the target device and its corresponding bandwidth. In addition, computation and loading are running in parallel. As the physical operation time occupies only a small part of the processing time and cannot be depicted, it is set as a constant. The processing time of each layer in a CNN can be indicated by:

(6) $$T^{\left(i\right)}=\max\left(T_{comp}^{\left(i\right)},T_{load}^{\left(i\right)}\right)+T_{po}^{\left(i\right)},$$

where,

(7) $$T_{comp}^{\left(i\right)}=F_{1}\left(N_{in}^{\left(i\right)},N_{out}^{\left(i\right)},S^{{\left(i\right)}^{2}},K^{{\left(i\right)}^{2}},1/T_{p}\right),$$

(8) $$T_{load}^{\left(i\right)}=F_{2}\left(IO^{\left(i\right)},1/Bandwidth\right),$$

(9) $$T_{po}^{\left(i\right)}=c.$$

In the above, the computation time of CNN is shown from a general perspective. However, due to different types of operations concerning each layer in CNN, their computation processes are also different, and the computation time of convolution layer $T_{conv}^{\left(i\right)}$, pooling layer $T_{pool}^{\left(i\right)}$ and fully connect layer $T_{fc}^{\left(i\right)}$ is expressed as:

(10) $$T_{conv}^{\left(i\right)}=\frac{\left\lfloor \frac{N_{in}^{\left(i\right)}}{PE_{num}}\right\rfloor \cdot N_{out}^{\left(i\right)}\cdot S^{{\left(i\right)}^{2}}\cdot K^{{\left(i\right)}^{2}}}{PE_{size}^{2}\cdot f\cdot T_{p}},$$

(11) $$T_{pool}^{\left(i\right)}=\frac{\left\lfloor \frac{N_{in}^{\left(i\right)}}{PE_{num}}\right\rfloor \cdot N_{out}^{\left(i\right)}\cdot {\left(\frac{R^{{\left(i\right)}^{2}}+padding}{2}\right)}^{2}\cdot K^{{\left(i\right)}^{2}}}{PE_{size}^{2}\cdot f\cdot T_{p}},$$

(12) $$T_{fc}^{\left(i\right)}=\frac{N_{in}^{\left(i\right)}\cdot N_{out}^{\left(i\right)}}{datain_{portnum}\cdot f\cdot T_{p}}.$$

For a CNN model *m* with *N-layer*, with the parallel mapping structure, its inference latency L(m) is the sum up of the processing time of each layer:

(13) $$L\left(m\right)=\sum_{i=1}^{N}T^{\left(i\right)}.$$

With the analysis on computing bottlenecks when a neural network is deployed on an edge device, and through the inference latency profiling model, we can obtain the inference latency of a single layer and the whole network. Therefore, it should be noticed that this inference latency profiling model is of great importance for us to construct the efficient NAS framework.

### 3.3. Device-Specific Profiling Model Evaluation

In this part, the accurateness of the proposed inference latency profiling model will be evaluated by comparing estimated results with actual measurements. Xilinx Zynq-7000 SoC ZC706 and Xilinx Virtex-7 FPGA VC707 were employed as the test devices, and their resources are shown in Table 2. The experiments were conducted by VGG-13 on the CIFAR-10 dataset.

Since in the proposed profiling model, the computation time and the loading time of each layer are affected by the data size, in order to evaluate the accurateness of the profiling model, the number of kernels in VGG-13 is adjusted to confirm the data size that should be processed. In experiments, *x*% kernels are randomly kept in VGG-13 (*x* from 100 to 10), which means the profiling model is evaluated under different 10 scenarios. Table 3 and Table 4 illustrate the comparison results. Then, it can be easily found that the profiling model possesses accuracies of 90.90–95.83%, with the average of 92.74%, which shows the high profiling accurateness.

## 4. Resource-Aware Bayesian Search for Edge Devices

Based on our proposed inference latency profiling model, we assign the Bayesian search with the ability of hardware resource awareness. In the search process, accuracy and inference latency are taken as the search targets. By finding the Pareto Optimal Front, the search direction is guided, and the CNN with a trade-off between accuracy and inference latency for the edge devices is also obtained. Moreover, since inference latency is the additional search target, the search space will be further narrowed down, and the search efficiency will also be enhanced.

The search process can be divided into two main stages. Firstly, the obtained CNNs are sorted and selected with accuracy and inference latency to update the search direction, which is the Bayesian Optimal process. Secondly, network morphism is performed according to the new search direction to obtain the new network structures. Our method is shown in Algorithm 1.
**Algorithm 1** Efficient Resource-aware Convolutional Neural Architecture Search with Pareto-Bayesian Optimization.1:**Input:** The initial CNN model *x*, the initial GP model M, the initial searched CNN queue S, the dataset D, the given search time T, the number of generated CNN in each round of search *N*  2:Push *x* into S  3:**while**T**do**4: **for** *x* in S **do**5:   *x* is trained with D  6:   Get the accuracy of *x*  7:   Get the latency of *x* with the profiling model  8: **end for** 9: S is sorted by $A^{*}$ search and finding the Pareto optimal front with accuracy and latency  10: M is fitted with the sorted S  11: **for** i in *N* **do**12:   Generate *x* with network morphism  13:   Push *x* into S  14:  **end for** 15:**end while** 16:**Output:** the CNN structure

### 4.1. The Kernel of Gaussian Process in Bayesian Search

Bayesian optimization is a model-based hyperparameter optimization method where a Gaussian process (GP) is adopted to update the posterior distribution of the objective function by continuously adding sample points without knowing the internal structure of the objective function that is usually defined as:

(14) $$X^{*}=\arg_{x\in S}\max f\left(x\right).$$

From the above equation, it can be seen that the goal of Bayesian optimization is to find *x* from the candidate set *S* to maximize f(x). When Bayesian optimization is applied for the purpose of searching for the CNN, each input *x* is a CNN structure, and its corresponding output f(x) is the accuracy and inference latency of this CNN structure. Here, it is assumed that f(x) satisfies the Gaussian distribution, with the prior distribution of f(x), and then new CNNs can be gradually added so as to update the distribution. These new CNNs are obtained from the previously generated network structure through network morphism, such as widening the layers and increasing the number of layers of the network. Subsequently, by continuously correcting and modifying the original assumed prior distribution according to the newly added CNNs, we can finally get the real distribution and the CNN with a trade-off between accuracy and inference latency that can be effectively deployed on the edge devices.

Furthermore, the GP is applied to update the Gaussian distribution. Additionally, in order to make Bayesian Optimization more suitable for network morphism, it is redesigned by [33]. In traditional Bayesian Optimization, the GP is usually adopted in the Euclidean space. However, the neural network architectures do not belong to Euclidean space and are difficult to parameterize into a fixed-length vector. It is impractical to directly vectorize neural networks. Therefore, an edit-distance based GP kernel is applied to calculate the operands of transforming an existing CNN to a new one.

Assuming $f_{a}$ and $f_{b}$ are two CNN structures, the kernel is defined as following:

(15) $$K\left(f_{a},f_{b}\right)=e^{-\rho^{2}\left(d\left(f_{a},f_{b}\right)\right)},$$

where d(·,·)∈[0,+∞) is the edit-distance between two CNN structures; ρ is a mapping function that maps the distance in the original metric space to the corresponding distance in the new space. Calculating the edit-distance of two neural networks can be seen as calculating the edit-distance of two graphs, and this approximate solution is denoted by:

(16) $$d\left(f_{a},f_{b}\right)=D_{l}\left(L_{a},L_{b}\right)+\lambda D_{s}\left(S_{a},S_{b}\right),$$

where $D_{l}$ is the edit-distance for morphing the layers and $D_{s}$ is for morphing the connections between layers. $L_{a}$ and $L_{b}$ are the set of layers of CNN $f_{a}$ and $f_{b}$, $S_{a}$ and $S_{b}$ are their layer connection sets. In addition, the $D_{l}$ and $D_{s}$ are calculated by:

(17) $$D_{l}\left(L_{a},L_{b}\right)=\min\sum_{i=1}^{\left|L_{a}\right|}d_{l}\left(l_{a}^{\left(i\right)},\varphi_{l}\left(l_{a}^{\left(i\right)}\right)\right)+\left|\right|L_{b}\left|-\right|L_{a}\left|\right|,$$

and

(18) $$D_{s}\left(S_{a},S_{b}\right)=\min\sum_{i=1}^{\left|S_{a}\right|}d_{s}\left(s_{a}^{\left(i\right)},\varphi_{s}\left(s_{a}^{\left(i\right)}\right)\right)+\left|\right|S_{b}\left|-\right|S_{a}\left|\right|,$$

where $\varphi_{l}$ and $\varphi_{s}$ are layer and connection injective functions, $d_{l}\left(\cdot ,\cdot \right)$ is the edit-distance of widening a layer into another, $d_{s}\left(\cdot ,\cdot \right)$ is the edit-distance for two matched connections.

Through the above kernel function, the GP model can be updated, and applied to guide the CNN generation in the next round of the search process. Then, the details of the CNN model selection will be given.

### 4.2. Pareto-Bayseian Search for Edge Computing

When searching for high accuracy and low latency CNNs for an edge device based on its computation resources, the search process is constrained from two dimensions. For a CNN structure, there is a conflict between its high accuracy and low latency. In order to get high accuracy, its structure will be deeper and wider. However, only with a limited scale of the structure, low latency can be achieved. Therefore, during our search process, these two searching objectives are optimized at the same time to guide the search direction, and finally, a CNN with a trade-off between accuracy and inference latency is obtained.

Two kinds of commonly-used multi-objective optimization methods are mainly shown below: (1) Convert the multi-objective problem into a mono-objective problem. How to combine different kinds of objectives and assign them with different weights is the key point to solve the optimization problem. (2) It is Pareto optimal. Compared with the above method, without manual intervention, the results of Pareto optimal are more objective and accurate. Therefore, in the search process, Pareto optimal is chosen to find the trade-off between accuracy and latency.

***Pareto optimal.*** In Pareto optimal, assume there are two objective functions f1 and f2: Dominant solution: the values of f1 and f2 belonging to solution *a* are better than those of solution *b*. In this case, solution *a* is superior to solution *b*, or in other words, solution *a* dominates solution *b*.

Non-dominant solution: If there are no other solutions better than solution *a*, that is, solution *a* is not dominated by other solutions, then solution *a* is called a non-dominant solution or the Pareto solution.

**Figure 3:**
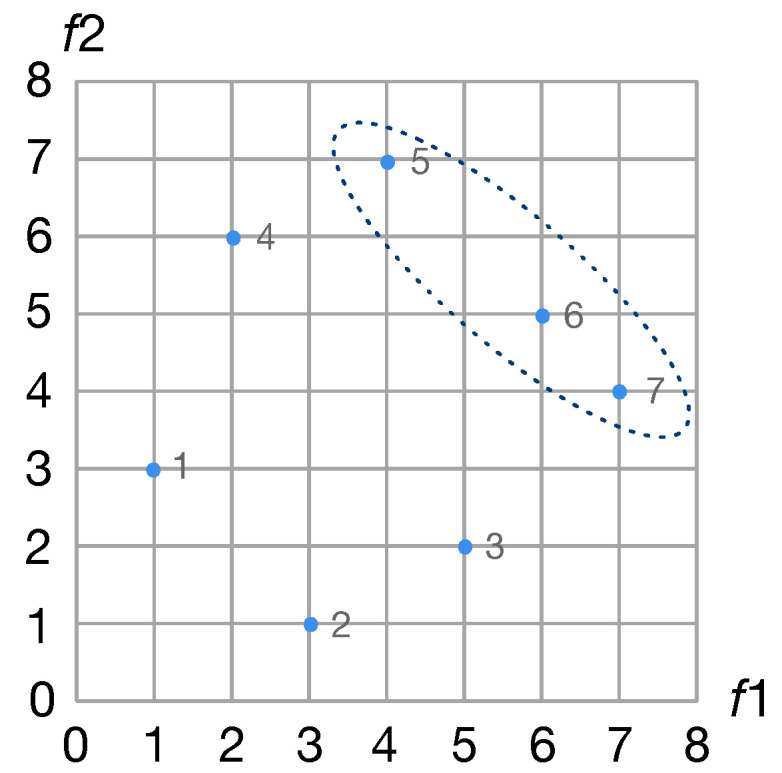
An example of the Pareto front. Solution 5, 6 and 7 are the non-dominant solutions, and they are the Pareto front.

Pareto front: the set of all non-dominant solutions. All the solutions in the Pareto front are not dominated by other solutions. There is an example of the Pareto front shown in Figure 3.

**Figure 4:**
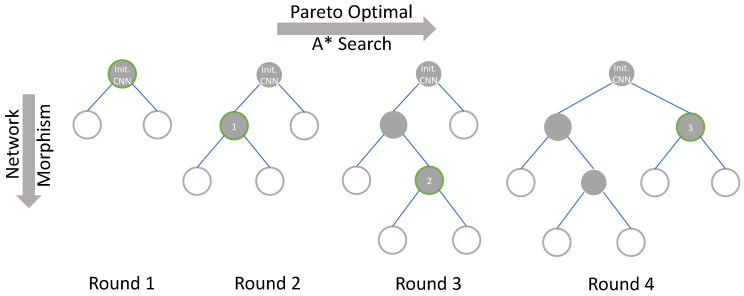
The tree-structured Pareto Bayesian search process. The gray node is the node that has been searched, the white node is the node that is known but not searched. The green border indicates that the node will perform network morphism in the current round to generate new network structures.

***Pareto-Bayesian search.*** In the Bayesian search, a tree-structured search process is applied as shown in Figure 4, starting with an initialized structure and expanding it, and all of its child nodes in the tree are the structures after the network morphism. The search process is to continuously select nodes in the tree and expand them, and finally obtain a network structure that meets the requirements. When applying the Pareto optimal to the search process, at each round of the search process, the new morphed networks are trained, and through the profiling model, their accuracy and inference latency are obtained. The accuracy and latency are saved as a tuple, by finding the Pareto front to get the results of Pareto optimal. Then, the Bayesian optimization is performed on the searched network structure in the Pareto front, and these networks are sorted by their accuracy and latency comprehensively. Apart from that, $A^{*}$[40] search is applied to determine which network structure will be morphed. $A^{*}$ search is an efficient tree-structure search algorithm. In the tree structure, it maintains a priority queue and can keep expanding the best node in the queue.

With $A^{*}$ search, for the searched networks, their priorities are obtained based on accuracy and latency and added to the priority queue. As the network with the highest priority is taken out and expanded by network morphism, then the priorities of the morphed networks are calculated. According to the acceptance function of the Simulated Annealing Algorithm, whether the new morphed networks are better than their father node is determined. If so, the new network is added to the top of the priority queue, and it is also considered as the starting point of the next network morphism.

It can be seen from Figure 4 that in the first round of search, the initialized CNN is subjected to morphism of the new CNNs according to the determined morphism direction with the GP model. Then, we can obtain the Pareto front of the accuracy and inference latency of these networks and sort them with the $A^{*}$ search. Furthermore, node 1 is selected after the first round of the search. Here, the structural features and morphism selections of node 1 will be applied to update the GP model. Since node 1 satisfies the high-accuracy and low-latency search objectives, in this round of network morphism, the changes in accuracy and resource consumptions caused by changes in network structure will be reflected in the Gaussian distribution and guide the subsequent network morphism. Thus, it is shown that when the search algorithm has hardware resource awareness, the search direction can be regulated. Later, in the second round of the search process, network morphism is performed on the basis of node 1, and the above operations are repeated. In addition, in each round of the search process, all the morphed CNNs are sorted, and each node has the opportunity to be selected for network morphism. Therefore, in round 4, node 3 is selected to be morphed rather than simply expand the child nodes of node 2. Through the above search method, by continuously evaluating the CNNs that have been selected and the new CNNs obtained by network morphism, we can more clearly know about the impact of operation selection and network structure expansion in the network morphism process on the accuracy and inference latency and have a trade-off between accuracy and inference latency. Based on this, the GP model can be updated, and the adaptive relationship between operation selections and resource consumptions will be recorded. After updating the GP model multiple times, it has a preference for network morphism to some extent. At this time, the GP model is adopted to get the direction of the network search for further guiding the search process more efficiently. In addition, the $A^{*}$ search can morph the networks from the nodes that are known but not searched, instead of only expanding the current optimal node, and the Simulated Annealing Algorithm assigns a probability mutation that is time-varying and tending to zero. The combination of these two methods can prevent the search process from falling into a local optimum.

Compared with the search process when only the accuracy is set as the search target, inference latency as an additional target avoids the brutal growth of the network structure in pursuit of higher accuracy, which is suppressing the growth of the tree structure and reducing the number of nodes to be searched in the tree structure. In addition to that, with two dimensions of search targets, the search direction is shrunk into a more narrowed range. Then, a clearer search direction can further help to reduce the number of networks that need to be evaluated. Therefore, the Pareto-Bayesian search can obtain a CNN with high accuracy and low latency, and it is more suitable to be deployed on the edge device and can further improve the search efficiency.

## 5. Parameter Saving Search Space

Due to the number of options when morphing the network in [33], the huge search space will greatly reduce search efficiency. In this part, the search space of the Bayesian search is optimized to enhance the search efficiency from the following two aspects: (1) The cell-based structure is applied to reduce the number of the options for further decreasing the amount of sampled networks during the search process, thereby, reducing the search space and improving the search efficiency; (2) the lightweight convolutional operation replaces the traditional convolutional operation to reduce the computation workloads, enhance the training efficiency during the search process and further improve the overall search efficiency.

In [33], there are four basic operations when network morphism: deep(G,u) adds a new layer after the $u_{th}$ layer; wide(G,u) widens the $u_{th}$ layer; add(G,u,v) adds an additive connection between the $u_{th}$ layer and the $v_{th}$ layer, and concat(G,u,v) adds a concatenative connection between the $u_{th}$ layer and the $v_{th}$ layer. When the network deepens, there are nine options for the new layer: Conv (1 × 1, 3 × 3, 5 × 5), Pooling (1 × 1, 3 × 3, 5 × 5), Relu, BatchNormalize (BN) and Dropout.

**Figure 5:**
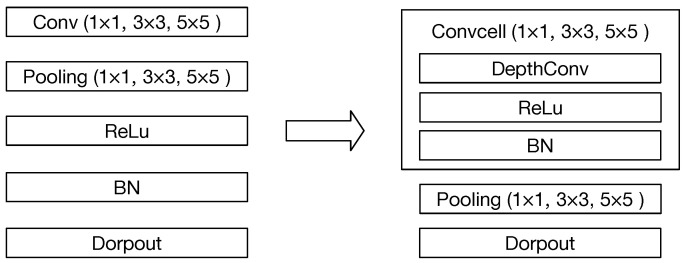
The cell-based convolutional operation.

At each round of the search process, although the Bayesian optimization can guide the search direction, there will be a large number of new sampled networks after the network morphism, which means a large number of networks are required to be trained to evaluate their accuracy and degrade the efficiency of the search process to a great extent. Therefore, the cell-based structure is applied to the convolutional operations shown in Figure 5. In this case, it can be seen that the number of options for the new layer is reduced from nine to seven, which will effectively reduce the number of sampled networks at each search stage, especially, when the network is deeper.

In order to further enhance the efficiency of the search process, the lightweight convolutional operation is employed. In this case, depthwise convolutional operation [41] is used to replace the traditional convolutional operation. Then, the computation workloads of these two operations with the same input are compared:

(19) $$\frac{CW_{DepthConv}}{CW_{Conv}}=\frac{M^{2}\cdot K^{2}\cdot C_{in}+M^{2}\cdot C_{in}\cdot C_{out}}{M^{2}\cdot K^{2}\cdot C_{in}\cdot C_{out}}=\frac{K^{2}+C_{out}}{K^{2}\cdot C_{out}}=\frac{1}{K^{2}}+\frac{1}{C_{out}},$$

where *M* is the input size; *K* is the kernel size; $C_{in}$ is the number of the input channel, and $C_{out}$ is the number of the output channel, when the computation workloads of depthwise convolutional operation are reduced significantly. Therefore, the reduction of the computation workloads will bring two benefits: (1) During the search process, the training process at each stage will be sped up, and the overall search efficiency will be improved; (2) when the searched CNN is deployed on the edge device, the inference latency will be further reduced.

## 6. Experiments

### 6.1. Implementation and Environment Details

Our experiments were implemented in TensorFlow with the 48-core Intel(R) Xeon(R) CPU E5-2650 v4 @ 2.20 GHz processor and an NVIDIA TESLA P100 GPU card. The MNIST dataset contains a training set of 60,000 images and a test set of 10,000 images with a dimension of 28 × 28 × 3. In the CIFAR-10 dataset, a training set of 50,000 images and a test set of 10,000 images with a dimension of 32 × 32 × 3 are comprised. The computing devices employed for edge computing are Xilinx Zynq-7000 SoC ZC706 and Xilinx Virtex-7 FPGA VC707, and their details are shown in Table 2. The initial architecture is a three-layer convolutional neural network with 64 filters and 3 × 3 Conv in each layer. During the search process, the stride is equal to one, and the number of filters equals 64.

### 6.2. Performance Evaluation

In this part, the experiments are conducted on the MNIST and CIFAR-10 datasets to evaluate compared with Bayesian search (BS), whether the Pareto Bayesian search (PBS) can obtain CNN with high accuracy and low latency that is more suitable to directly deploy on the edge devices. There are three search time settings, and the results are shown in Table 5 and Table 6. The results demonstrate that the PBS can effectively search for the CNN with a trade-off between accuracy and inference latency.

Under the three experimental settings, for the target device ZC706, on the MNIST dataset, compared with BS, the accuracy variation is −0.21%, −0.16%, and −0.21%, and the inference latency is reduced by 92.43%, 94.71%, and 94.66%. On the CIFAR-10 dataset, the accuracy variation is −2.20%, −1.51% and, −1.09%, while the inference latency is reduced by 87.44%, 90.59%, and 90.36%. For VC707, the accuracy variation is −0.17%, −0.17%, and −0.13%, and the inference latency is reduced by 90.57%, 92.40%, and 92.95%, respectively, on MNIST. Furthermore, on CIFAR-10, the accuracy variation is −1.44%, −1.89% and, −1.60%, and the inference latency is reduced by 90.41%, 92.91%, and 93.89%, respectively. The CNN searched by PBS gains a huge benefit on the inference latency only by sacrificing a tiny of accuracy. Therefore, PBS can search for a better CNN than BS for edge computing.

**Figure 6:**
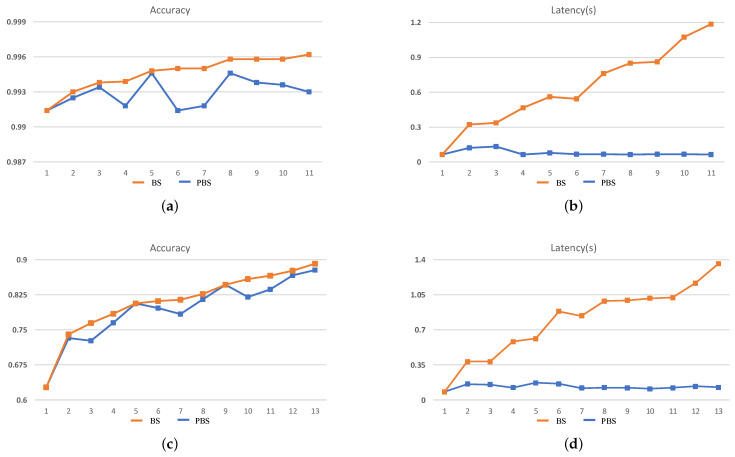
The search results for ZC706. (**a**,**b**) The accuracy and inference latency of the searched networks on MNIST and (**c**,**d**) the CIFAR-10 within 240 min.

**Figure 7:**
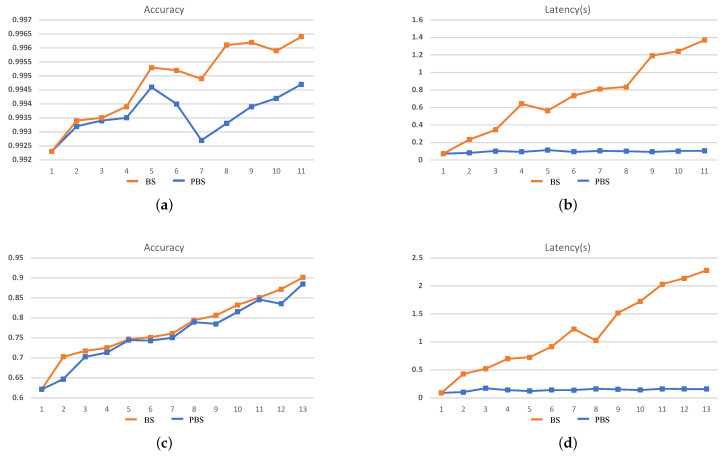
The search results for VC707. (**a**,**b**) The accuracy and inference latency of the searched networks on MNIST and (**c**,**d**) the CIFAR-10 within 240 min.

The search results cannot fully demonstrate the efficiency of the search process. Therefore, in order to illustrate that the two dimensions of search objectives in PBS can effectively guide the search direction, the intermediate results of each stage during the search process should be paid more attention to. In this case, we take the 240-min setting as an example for analysis, and the search results are shown in Figure 6 and Figure 7.

It can be seen that during the BS search process, accuracy is the mono-objective to guide the search process and ignores the increase of inference latency brought by an increase in the scale of the network structure. As a result, with the continuous increase in the accuracy of the searched CNN at each stage, the inference latency is also increased, which demonstrates that during the search process, at each stage, the new generated CNNs are morphed from the search result of the last stage, which means in the tree-structured search space, the BS tries to evaluate the deeper leaf nodes and will result in an explosive growth of the search space. On the contrary, during the PBS search process, although the accuracy of the searched CNN is gradually increasing, the inference latency eventually only changes within a small range. The latency std of the searched network is only 0.024 and 0.028 on MNIST and CIFAR-10, respectively, for ZC706 in Figure 6. For VC707, the std is 0.012 and 0.023 on MNIST and CIFAR-10 accordingly in Figure 7. The comparison with the search results in Figure 2 indicates the search process is looking for a trade-off between accuracy and inference latency and constantly searching for the generated but not searched nodes in the tree-structured search space. With two dimensions of search targets, the search space will not explosively grow as the BS and has the clear trending of convergence. At the same time, the continuous increase of the accuracy and the eventual concentrate of the inference latency demonstrate that the PBS can effectively guide the search direction, and prevent the search process from falling into a local optimum.

The above results reveal that our proposed method can effectively regulate the search direction to search for networks with high accuracy and low inference latency. Furthermore, the search time is enlarged to search for the network with higher accuracy. Then, two mono-objective NAS methods (they only set accuracy of the network as the search objective) and two multi-objective NAS methods (they also set accuracy and inference latency of the network as the search objective, which is the same as our method) are selected as the SOTA method for comparison. Among them, ENAS [24] as an efficient mono-objective NAS method, can search for a high-accuracy network structure within 0.4 GPU days. Therefore, the search time is set to 0.4 GPU days here. The search results are shown in Table 7. Compared with the two SOTA mono-objective search methods, the accuracy of the network structure searched for ZC706 by our method is reduced by 0.28% and 0.88%, and for VC707 is reduced by 0.16% and 0.76%. Compared with the other two multi-objective search methods, the accuracy is enhanced by 0.13% and 7.55% for ZC706, 0.30% and 7.67% for VC707. In the mono-objective search process, the searched network structures can become deeper and wider arbitrarily, while, our method constrains the search direction and the network structure to a certain extent. In this case, the NAS [19] and ENAS [24] can search for networks with higher accuracy. Compared with other multi-objective search algorithms, our method has more advantages and can obtain networks with higher accuracy within a more limited search time. At the same time, the network structure searched on the CIFAR-10 is transferred to the ImageNet dataset, and the Top 1 accuracy of 64.87% and 65.23%, respectively, can be obtained. The results of transfer learning demonstrate the application potentials of our method.

### 6.3. Efficiency Evaluation

In this part, the ability of optimized search structure and space to improve the overall search efficiency will be evaluated. In the previous section, the reduction of options for the new layer with the cell-based convolutional operations that can reduce the search space has been illustrated. Since the optimized search space is still very huge, it is impossible to evaluate the improvement of overall search efficiency with a thorough search. Therefore, in this part, only the improvements brought by the lightweight operation are experimentally evaluated. The efficiency of PBS and lightweight PBS is compared by the number of search stages within the same search time. The experiments are conducted for ZC706 on five different search time settings, and the results are shown in Table 8 and Table 9. It can be seen that when the search time is 150 min, on MNIST, the number of search stages is four and two for Lightweight PBS and PBS, respectively. The search efficiency is increased by 100%. Furthermore, on CIFAR-10, the search efficiency is increased by 150%. However, due to the short search time and too few networks searched, the network structures differ greatly. Thus, the training time is extremely different, which is not enough to fully reflect the improvement of search efficiency of Lightweight PBS. When the search time gradually increases and accuracy and inference latency are taken as the two-dimensional constraints to regulate the search direction, the search process will focus more on the choice of computing operations. At this time, it can better reflect the improvement of search efficiency by Lightweight PBS. When the search time is longer than 210 min, an 18.18% search efficiency improvement can be obtained on MNIST at most, and 7.7% improvement on CIFAR-10. The results show that under five settings, the lightweight PBS can always search more CNNs. With the lightweight operation, the time cost of training at each search stage is reduced to enhance the search efficiency.

**Figure 8:**
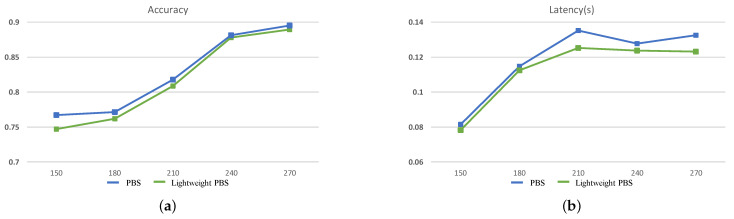
The search results on CIFAR-10 in 5 different search time settings. (**a**) is the accuracy of the searched networks, (**b**) is the latency of them.

**Figure 9:**
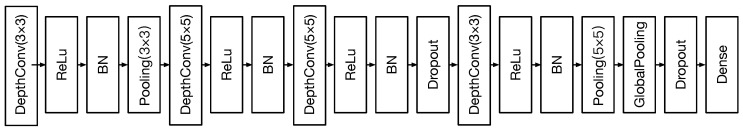
The searched CNN for MNIST.

**Figure 10:**
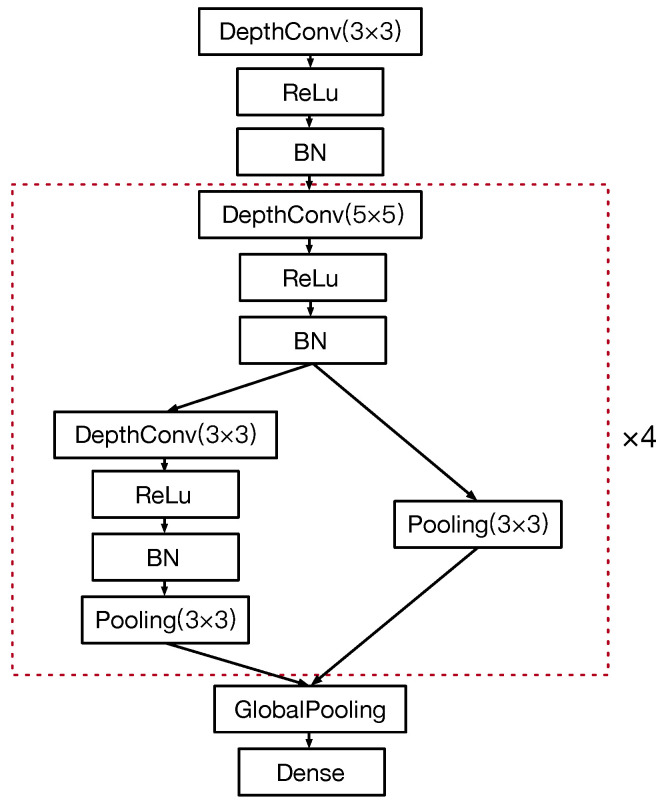
The searched CNN for CIFAR-10.

Besides what is mentioned above, the ability of lightweight PBS to search for the better CNN is also evaluated. The accuracy and inference latency of the searched optimal CNN under different search time settings are shown in Figure 8. With different search times, the accuracy of the searched CNNs when applying the lightweight convolutional operation can be compared or even surpass the results of the PBS. At the same time, when the scale of the searched CNNs is similar, with the lightweight operation, the inference latency will be smaller. Furthermore, with the increase of the search time, the inference latency is not continuously increased, and the guidelines for the search direction of our method are demonstrated once again. The best CNNs for MNIST and CIFAR-10 datasets are shown in Figure 9 and Figure 10. In summary, the Pareto Bayesian search and optimized search structure can help us obtain a CNN more efficiently with a trade-off between higher accuracy and lower latency for edge computing.

## 7. Conclusions and Future Work

In this study, in order to obtain the CNN structure efficiently deployed on the resource-constrained edge devices, an efficient resource-aware convolutional neural network search method is proposed. During the search process, the proposed profiling model is adopted to be aware of the resources of the edge device and then obtain the inference latency of the CNN structure, further combined with the accuracy of the CNN as the two-dimensional targets for the CNN search process. In each stage of the Bayesian search, the trade-off between accuracy and inference latency is obtained by finding the Pareto front, and the search direction is narrowed down to a smaller range that can more efficiently search for the CNN with a trade-off between high accuracy and low latency. At the same time, through the cell-based search structure, the search space is reduced to improve search efficiency, and the lightweight convolutional operations are applied to reduce the time cost of training in each search stage and the inference latency of the obtained CNN. Finally, from the experimental results, it can be found that the proposed method can help us efficiently obtain a CNN structure with high accuracy and low latency that is more suitable for deploying on an edge device.

In the future, further research will be conducted on two aspects. Firstly, for the search objectives, in this paper, only the accuracy and inference latency of the network are considered as the search objectives. In future work, more hardware performances, such as the power consumption and energy consumption of the network deployed on an edge device will be taken as the search objectives, and whether higher dimensional constraints will further improve the search efficiency will also be verified. Secondly, the search algorithm will be designed directly for the image with a larger size. In this article, our method is certified on the MNIST and CIFAR-10, and the networks searched on CIFAR-10 are further transferred to the ImageNet for verification. The experiment results demonstrate that our searched networks have a competitive performance. In the future study, the challenges when searching for large-size images and the way of enhancing the efficiency of the search process will be further focused on.

## Figures and Tables

**Table 1:** Description of notations.

Notations	Description
$R^{{\left(i\right)}^{2}}$	The size of Input data of ith layer
$N_{in}^{\left(i\right)}$	Input feature map number of the ith layer
$S^{{\left(i\right)}^{2}}$	The size of output data of ith layer
$N_{out}^{\left(i\right)}$	Output feature map number of the ith layer
$K^{{\left(i\right)}^{2}}$	Kernel size
$N_{in}^{\left(i\right)}\cdot N_{out}^{\left(i\right)}$	The number of kernels
${IO}^{\left(i\right)}$	Date movement
bitwidth	Data bitwidth
bandwidth	Bandwidth
PE	Process Element
$PE_{num}$	The number of PE
$PE_{size}$	The size of PE
*f*	Clock frequency
paddinig	The width of padding
$datain_{portnum}$	The number of input data shifts

**Table 2:** Resources of the selected test devices.

Type	Xilinx Zynq-7000 SoC ZC706	Xilinx Virtex-7 FPGA VC707
LUT	350	485,760
Block RAM (Mb)	19.1	37.08
DSP slice	900	2800
Bandwidth(Gbps)	9.6	1.6

**Table 3:** Profiling accurateness evaluation for *VGG-13* on ZC706.

Ratios%	100	90	80	70	60	50	40	30	20	10
**Est (ms).**	1129.24	1009.32	905.24	782.36	678.38	566.29	452.12	339.45	225.59	112.48
**Act (ms).**	1178.37	1056.29	966.47	851.74	746.23	602.19	496.87	370.27	239.17	122.37

**Table 4:** Profiling accurateness evaluation for *VGG-13* on VC707.

Ratios%	100	90	80	70	60	50	40	30	20	10
**Est (ms).**	1436.49	1246.83	1148.14	941.78	896.52	691.94	577.94	408.81	266.69	168.83
**Act (ms).**	1523.02	1357.91	1251.79	1013.65	853.31	765.34	618.45	442.53	291.24	186.51

**Table 5:** The search results on MNIST.

Search Time	Performance	ZC706			VC707		
		BS	PBS	Variation (%)	BS	PBS	Variation (%)
210 (min)	Accuracy (%)	99.58	99.37	−0.21%	99.51	99.34	−0.17%
	Latency (ms)	862.21	65.21	**−92.43%**	1042.15	98.27	**−90.57%**
240 (min)	Accuracy (%)	99.62	99.46	−0.16%	99.64	99.47	−0.17%
	Latency (ms)	1185.32	62.66	**−94.71%**	1369.52	104.12	**−92.40%**
270 (min)	Accuracy (%)	99.67	99.49	−0.18%	99.59	99.46	−0.13%
	Latency (ms)	1213.40	64.74	**−94.66%**	1497.94	105.54	**−92.95%**

**Table 6:** The search results on CIFAR-10.

Search Time	Performance	ZC706			VC707		
		BS	PBS	Variation (%)	BS	PBS	Variation (%)
210 (min)	Accuracy (%)	82.66	80.84	−2.20%	83.07	81.63	−1.44
	Latency (ms)	986.15	123.81	**−87.44%**	1666.32	159.80	**−90.41%**
240 (min)	Accuracy (%)	89.16	87.81	−1.51%	88.92	87.03	−1.89%
	Latency (ms)	1358.74	127.76	**−90.59%**	2278.11	161.51	**−92.91%**
270 (min)	Accuracy (%)	89.23	88.25	−1.09%	90.12	88.52	−1.60%
	Latency (ms)	1412.14	136.15	**−90.36%**	2630.93	160.75	**−93.89%**

**Table 7:** Comparison with other methods.

Method	Dateset	Accuracy (%)	Search Time (GPU Days)
NAS [19]	CIFAR-10	95.53	22,400
ENAS [24]	CIFAR-10	96.13	0.4
DPPNet-ES [23]	CIFAR-10	95.07	8
FBNet [42,43]	CIFAR-10	87.7	9
Ours (ZC706)	CIFAR-10	95.25	0.4
Ours (VC707)	CIFAR-10	95.37	0.4
Ours (ZC706)	ImageNet (Transfer)	64.87 (Top 1)	0.4
Ours (VC707)	ImageNet (Transfer)	65.23 (Top 1)	0.4

**Table 8:** The number of stages during the search process on MNIST.

	150 (min)	180 (min)	210 (min)	240 (min)	270 (min)
PBS	2	5	9	11	12
Lightweight PBS	4	6	9	13	14

**Table 9:** The number of stages during the search process on CIFAR-10.

	150 (min)	180 (min)	210 (min)	240 (min)	270 (min)
PBS	2	4	8	13	13
Lightweight PBS	5	7	7	13	14
